# The prognostic value of baseline EARL standardized FDG PET indices in pediatric and adolescent high-grade osteosarcoma

**DOI:** 10.1007/s00330-025-11372-z

**Published:** 2025-01-24

**Authors:** Roelof van Ewijk, Rana Dandis, Janna Rodewijk, Bart de Keizer, Simone A. J. ter Horst, Michiel A. J. van de Sande, Lizz van der Heijden, Johannes H. M. Merks, Lianne M. Haveman, Arthur J. A. T. Braat

**Affiliations:** 1 Princess Máxima Center for Pediatric Oncology, Utrecht, The Netherlands; 2 Division of Imaging and Oncology, University Medical Center Utrecht, Utrecht University, Utrecht, The Netherlands; 3 Department of Nuclear Medicine, Netherlands Cancer Institute, Amsterdam, The Netherlands

**Keywords:** Osteosarcoma, Pediatrics, PET-CT, Prognostic factors, Quality control.

## Abstract

**Objective:**

To investigate the prognostic value of baseline European Association of Nuclear Medicine Research Ltd. (EARL) standardized [^18^F]fluorodeoxyglucose positron emission tomography-computed tomography ([^18^F]FDG PET-CT) quantitative values for survival and to evaluate cutoff values identified in other studies.

**Materials and methods:**

Pediatric and adolescent patients with high-grade osteosarcoma were included. Baseline [^18^F]FDG PET-CT, with EARL-accredited reconstructions, was the standard diagnostic staging procedure. Cox proportional hazard analysis for event-free survival (EFS) and overall survival (OS) was performed with clinical prognostic factors. Kaplan–Meier analysis and log-rank tests were applied to investigate the prognostic performance of the [^18^F]FDG PET indices.

**Results:**

In total, 66 patients were included in this study. In the univariable Cox regression analysis, peak lean-body mass corrected SUV (SUL_peak_) (hazard ratio (HR): 1.04), total lesion glycolysis (TLG) (HR: 1.0), and metabolic tumor volume (MTV) (HR: 1.0) were not associated with EFS or OS. Log-rank analysis showed a significant difference in EFS for all SUL_max_ and SUL_peak_ cutoffs. For MTV_total_ the maximum Youden, and for TLG_total,_ the maximum Youden and maximally selected rank cutoff resulted in a significant EFS difference. No cutoff for any measure showed a significant difference in OS between the groups. ROC curves for event status had an AUC of 0.67, 0.66, 0.64, and 0.64 for SUL_max_, SUL_peak_, MTV_total,_ and TLG_total_, respectively.

**Conclusion:**

In this study, the baseline EARL-standardized [^18^F]FDG PET indices of children and adolescents with osteosarcoma were not prognostic of EFS or OS. The proposed prognostic cutoffs from earlier studies suffer from important technical and statistical issues.

**Key Points:**

***Question*** *Prognostic value of baseline [*^*18*^*F]FDG PET-CT imaging markers have been reported for histologic response and survival in high-grade osteosarcoma but have not been validated for clinical practice.*

***Findings*** *Baseline SUV*_*peak*_, *TLG*_*total*_*, and MTV*_*total*_ *measured on EARL-accredited reconstructions were not prognostic factors for survival in pediatric and adolescent patients with high-grade osteosarcoma.*

***Clinical relevance*** *A wide range of values for SUV*_*peak*_ *and SUV*_*max*_ *cutoffs with similar prognostic value were identified, questioning the value of a single proposed cutoff. Lack of validation, with important technical and statistical issues of proposed prognostic cutoffs, limits clinical implementation.*

## Introduction

High-grade osteosarcoma is the most common primary malignant bone tumor in children and adolescents, although it is rare in absolute numbers [1–3]. Despite multi-agent chemotherapy and aggressive surgery, survival remains poor, with a 5-year event-free survival (EFS) of 65% in localized disease and 30% in metastatic disease [4, 5].

At diagnosis, age, tumor size, tumor site, and disease stage are known prognostic markers [6]. At resection, surgical margins and histologic response to neoadjuvant chemotherapy have been shown to be important prognostic factors. The risk of recurrence or progression of disease more than doubles in patients with a poor response to chemotherapy [4, 7, 8]. Improved stratification could support the selection of patients in need of new therapeutic approaches and those eligible for treatment de-intensification, as current chemotherapy strategies have important late effects such as cardiotoxicity, ototoxicity, nephrotoxicity, and the risk of secondary malignancies [9–12].

Whole-body [^18^F]fluorodeoxyglucose positron emission tomography-computed tomography ([^18^F]FDG PET-CT) is increasingly utilized for disease staging [13–15]. Multiple [^18^F]FDG PET-CT-derived imaging markers, including peak and maximum standardized uptake value (SUV_peak_, SUV_max_) and metabolic tumor volume (MTV) at baseline, have been reported to be associated with histologic response and survival in mostly retrospective, mixed (bone) sarcoma studies, including a limited number of patients [16–27]. In a prospective, hypothesis-generating study by Im et al including 34 patients, SUV_peak_, MTV, and total lesion glycolysis (TLG) were predictive of EFS and overall survival (OS) at baseline, interim assessment, and post-therapy [16]. In a retrospective study by Palmerini et al, including 77 patients with bone sarcoma (32 osteosarcoma), SUV_max_ was a prognostic factor for EFS, with an applied cutoff of 6 [18]. As technical and measurement variability is known to influence quantitative measurements, the question is how to interpret the provided cutoff values for clinical practice [28–31], as reconstructions were commonly not based on international quality standards, such as the guidelines developed in Europe by the European Association of Nuclear Medicine (EANM) and the European Association of Nuclear Medicine Research Ltd. (EARL) [28, 32, 33] and in North America by the Quantitative Imaging Biomarkers Alliance (QIBA) and National Cancer Institute (NCI) [29, 30].

The primary aim of our study was to investigate the prognostic value for EFS and OS of [^18^F]FDG PET-CT quantitative values at diagnosis in patients with high-grade osteosarcoma treated in a single center with the availability of EARL-accredited [^18^F]FDG PET reconstructions. The secondary aim was to evaluate the proposed [^18^F]FDG PET-CT quantitative cutoffs, as identified in published studies [16–18].

## Materials and methods

### Participant selection

Pediatric and adolescent patients up to 18 years of age diagnosed with resectable high-grade osteosarcoma were included at a single center for pediatric oncology. Inclusion criteria were: (1) diagnosis between the 1^st^ of January 2018 and the 1^st^ of December 2022; (2) treatment with multi-agent chemotherapy according to the standard arm of the Euramos protocol [7, 8]; (3) a baseline [^18^F]FDG PET-CT performed according to EARL/EANM guidelines [34]. Data were retrospectively collected as part of the biobank and data access initiative of the institute, for which all included patients and/or their legal representatives provided signed informed consent. The Institutional Medical Ethics Review Board confirmed that the Medical Research Involving Human Subjects Act (WMO) did not apply to the study (research protocol number 23-207/DB).

### Image acquisition and reconstruction

PET-CT was performed using a Biograph VISION 600 (Siemens), after 6 h of fasting. Additionally, one hour before [^18^F]FDG injection, oral propranolol was administered (20 mg once one hour prior to FDG injection) to suppress brown fat tissue activation [33]. Image acquisition was performed one hour after the injection of 2.0 MBq/kg [^18^F]FDG. Images were reconstructed using an EARL-accredited protocol (version 1.0). First, a low-dose CT scan was acquired with an automatic tube voltage selection and current modulation (reference: 100 kV, 20 mAs) using CARE kV and CARE Dose4D (Siemens Healthcare). Low-dose CT was used for attenuation correction. Total body scan was performed using continuous bed motion, scanning two different speeds; 1.6 mm/s for head to pelvis and 3.2 mm/s for lower limbs (approx. 2 min/bed position for head to pelvis and 1 min/bed position for the legs). PET images were reconstructed using point spread function and time of flight modeling, four iterations with five subsets, and 4-mm Gaussian filtering. Image reconstruction matrix was 440 × 440 resulting in 1.65 × 1.65 mm pixels. PET images were reconstructed to a slice thickness of 3 mm [34].

### Image analysis

Quantification of the [^18^F]FDG PET-CT images was performed using a commercially semi-automated segmentation algorithm (Syngo.via), by liver activity-based thresholding for volume of interest (VOI) delineation, and SUV was lean-body mass corrected (SUL), in line with Positron Emission Tomography Response Criteria in Solid Tumors (PERCIST) [35]. SUL_max_, SUL_peak_, MTV, and TLG were measured in the primary tumor and the most avid lymph node, bone, and/or lung metastases, respectively. Additionally, the patient-based total tumor load, consisting of TLG and MTV of all pathologic lesions combined (TLG_total_ and MTV_total_), was collected. Semi-automated measurements of [^18^F]FDG PET-CT were checked by two expert nuclear physicians (AB/BK; > 10 years of experience). In addition, the maximum tumor diameter was measured using the corresponding low-dose CT.

### Statistical analysis

The distribution of the clinical characteristics was summarized using descriptive statistics. Differences between cohorts were compared using the chi-square or Fisher’s exact test, depending on the frequency distribution of each variable. The mean values of the [^18^F]FDG PET-CT variables were compared between cohorts using independent *t*-tests. Survival probabilities were estimated using Kaplan–Meier analysis. Event-free survival (EFS) was defined as the time from the date of diagnosis to the time of the first event, defined as death from any cause, disease progression, or relapse after previous complete remission. Overall survival (OS) was defined as the time from the date of diagnosis to death from any cause or the time of the latest follow-up. A Cox proportional hazard regression model was used to estimate the association between SUL_peak_, MTV, TLG, disease stage (localized/metastatic), histological response (≤/> 10% vital tumor), tumor size (</≥ 8 cm), and survival (EFS/OS). SUL_max_ was excluded from the Cox proportional regression analysis because of collinearity with SUL_peak_. [^18^F]FDG PET-CT indices were separately analyzed in multivariable analysis along with disease stage and histologic response. For continuous [^18^F]FDG PET values, the proportional hazard assumption was checked by graphical analysis and testing based on scaled Schoenfeld residuals. To assess linearity, the functional form of the relationship between [^18^F]FDG PET-CT indices and EFS was examined. This was performed using Martingale residual plots [36] with low smoothness, which displays an excess risk of progression or death (*y*-axis) in relation to the continuous covariate (*x*-axis). The optimal cutoff value was estimated using maximally selected rank statistics [37]. By evaluating various potential cutoff values for the indices and at each cutoff, examining the difference in survival between the groups formed by the cutoff, we identified the cutoff value that maximized the statistical significance of this difference as the optimal cutoff.

The log-rank test was used to test the survival probability curves based on different cutoffs for SUL_max_, SUL_peak_, MTV, and TLG:  (1) the median value of our cohort; (2) the optimal cutoff value as determined by the maximum Youden index from receiver operating characteristic analysis for events as applied by previous studies [16, 38]; (3) the optimal cutoff value estimated using maximally selected rank statistics; (4) based on indices from Im et al [16] and Palmerini et al [18]. The area under the curve (AUC) was calculated from the receiver operator curves (ROC curves) for SUL_max_, SUL_peak_, MTV, TLG, and event status. All statistical analyses were performed using R software version 4.1.1 [39].

### Reporting

This study is reported according to the STrengthening the Reporting of Observational Studies in Epidemiology (STROBE) guidelines [40].

## Results

### Patient characteristics

**Fig. 1:**
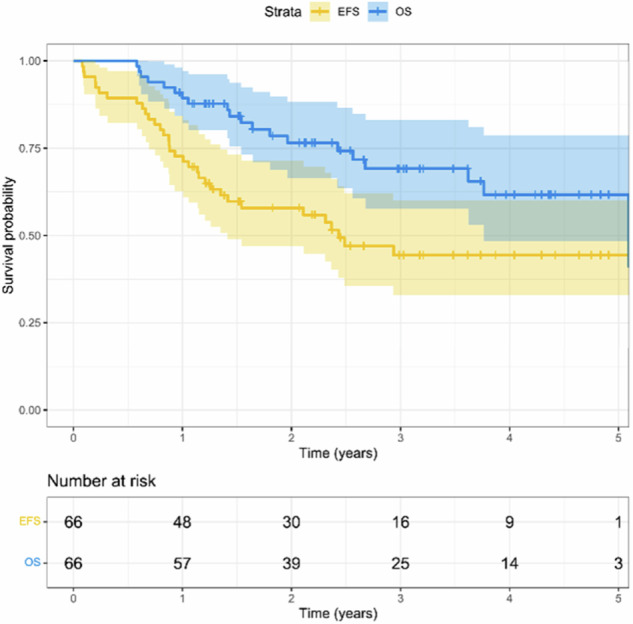
Event-free survival (EFS) and overall survival (OS) for included cohort

Between 2018 and 2022, 66 patients were included in this study (Table 1). Tumors were primarily located in the limbs, mainly in the lower extremity (*n* = 60, 91%), followed by the upper extremity (*n* = 5, 8%), and one axial pelvic tumor (*n* = 1, 2%). Metastatic disease at diagnosis was present in 21 (32%) patients. A poor histologic response to neoadjuvant chemotherapy (> 10% of vital tumor) was observed in 64% of patients. Four patients were non-evaluable (6%) for histologic response: one patient had primary surgery at diagnosis, and three metastatic patients did not undergo surgery due to early progression of disease. With a median follow-up of 28.8 months (range 7.0–63.7), 33 events were observed. In total, 20 patients died (17 due to disease progression and 3 due to toxicity). The 3-year EFS and OS rates were 44 and 69%, respectively (Fig. 1).

**Table 1:** Patient, tumor, and treatment characteristics

	Overall (*N* = 66)
Age (years)	
Median (Min, Max)	14 (4.0, 18.0)
Gender	
Male	42 (64%)
Female	24 (36%)
Primary tumor site	
Lower extremity	60 (91%)
Upper extremity	5 (8%)
Axial	1 (2%)
Primary tumor size	
< 8 cm	12 (18%)
≥ 8 cm	54 (82%)
Skip lesions	
Yes	3 (5%)
Pathologic fracture	
Yes	2 (3%)
Disease stage	
Localized	45 (68%)
Metastatic	21 (32%)
*Pulmonary only*	15 (23%)
Histologic response	
Good	20 (30%)
Poor	42 (64%)
Not available	4 (6%)

### [^18^F]FDG PET-CT

The mean glucose level was 5.0 mmol/L (SD 0.7 mmol/L). Propranolol was administered to 57 (86%) patients. Mean SUL_max_ was 9.14 (SD 4.3), mean SUL_peak_ 7.62 (SD 3.6), mean TLG_total_ 589 (SD 434), and MTV_total_ 146 cm^3^ (SD 98.5 cm^3^). The TLG and MTV of the primary tumor were 587 (SD 433) and 145 cm^3^ (SD 97.9 cm^3^), respectively. Of the known metastatic lesions, 12 of the 21 (57%) metastatic patients had non-avid pulmonary metastases, either due to a partial volume effect or lesions under the defined blood pool- or liver-derived PERCIST threshold. Evaluation of mean SUL_max,_ SUL_peak_, MTV_total_, and TLG_total_ for patients with a good (≤ 10% vital cells) vs. poor histological response (Supplemental Tables S1 and S2), and for patients with or without an event (Supplemental Table S3), did not show any significant differences between subgroups.

### Cox regression analysis

**Fig. 2:**
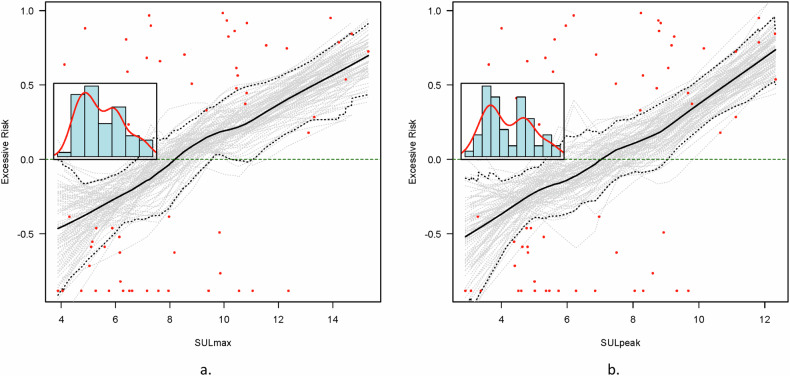
Martingale residual plot with lowess smooth, which displays excessive risk for event-free survival (*y*-axis) in relation to the continuous covariate (*x*-axis) data (**a** SUL_max_, **b** SUL_peak_). Both parameters show a linear relation between the increase of the parameter and the excessive risk for an event

**Fig. 3:**
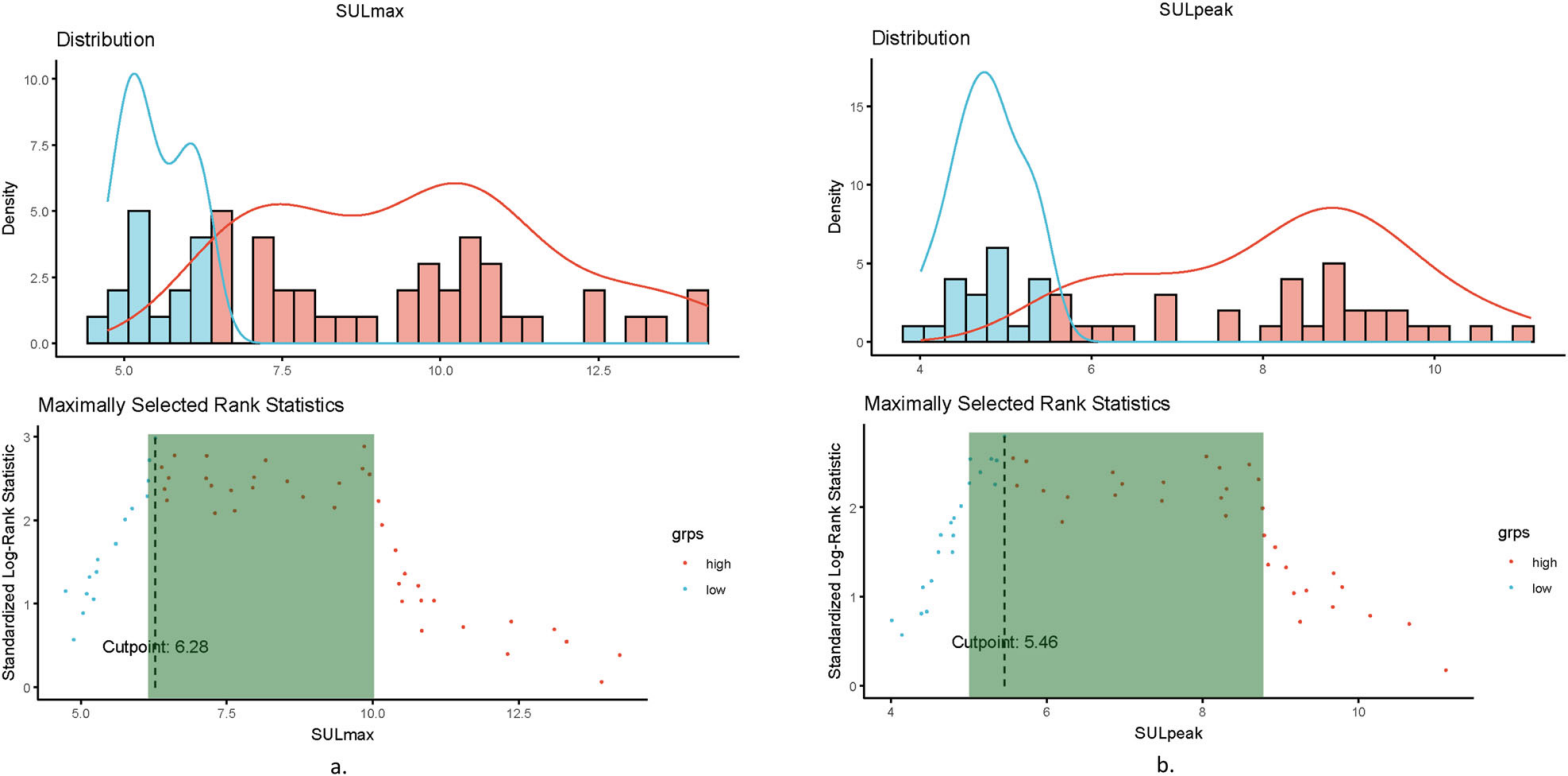
**a** Distribution by class (blue < cutoff vs. red > cutoff point) and standardized log-rank statistic for maximally selected rank statistics for SUL_max_. The optimal cutoff point for this cohort is 6.28. The standardized log-rank statistic is in a similar range for SUL_max_ values between 6 to 10, implying equal performance for any cutoff selected between these values (represented in the gray box). **b** Distribution by class (blue < cutoff vs. red > cutoff point) and standardized log-rank statistic for maximally selected rank statistics for SUL_peak_. The optimal cutoff point for this cohort is 5.46. The standardized log-rank statistic is in a similar range for SUL_peak_ values between 5 to 9, implying equal performance for any cutoff selected between these values (represented in the gray box)

The proportional hazard assumption was met, as there was no statistically significant relationship between the scaled Schoenfield residuals and time. The linearity assumption of the indices was investigated visually using Martingale residual plots (Fig. 2 and Supplemental Fig. S1). The optimal cutoff value was estimated using maximally selected rank statistics (Fig. 3 and Supplemental Fig. S2).

In univariable analysis, SUL_max_, SUL_peak_, TLG_total_, and MTV_total_ were not associated with EFS or OS (Table 2). Disease stage (localized vs. metastatic) was significantly associated with EFS (hazard ratio (HR) 2.2, 95% confidence interval (CI) 1.2–4.5) but not with OS (HR 1.6, 95% CI 0.65–4.1). Tumor size and histologic response were not associated with EFS or OS, despite borderline significance for histologic response in multivariable Cox regression (Table 3), which had a limited effect size and large CI.

**Table 2:** Univariable Cox regression analysis for event-free (EFS) and overall survival (OS)

	HR (EFS)	(95% CI for HR)	*p-*value	HR (OS)	(95% CI for HR)	*p*-value
SUL_max_	1.04	(0.97–1.11)	0.31	1.0	(0.9–1.11)	0.97
SUL_peak_	1.04	(0.96–1.13)	0.34	1.0	(0.88–1.12)	0.90
TLG_total_	1.0	(1.0–1.0)	0.26	1.0	(1.0–1.0)	0.84
MTV_total_	1.0	(1.0–1.01)	0.16	1.0	(1.0–1.01)	0.42
Staging (localized vs. metastatic)	2.2	(1.2–4.5)	0.03^a^	1.6	(0.65–4.1)	0.29
Tumor size (</≥ 8 cm)	2.4	(0.74–7.9)	0.15	3.4	(0.45–25.3)	0.24
Histologic response (good/poor)	1.9	(0.8–4.4)	0.15	2.1	(0.59–7.3)	0.26

*CI* confidence interval, *EFS* event-free survival, *HR* hazard ratio, *MTV* metabolic tumor volume, *SUL* standardized uptake value lean-body mass corrected, *TLG* total lesion glycolysis, *OS* overall survival

^a^ Indicates significance

**Table 3:** Multivariable Cox regression analysis for event-free survival

	HR (EFS)	(95% CI for HR)	*p*-value	HR (OS)	(95% CI for HR)	*p-*value
SUL_peak_	1.0	(1.0–1.1)	0.36	1.0	(0.9–1.1)	0.9
Staging (metastatic vs. localized)	2.7	(1.2–6.0)	0.02^a^	1.8	(0.6–5.4)	0.31
Histologic response (poor vs. good)	2.5	(1.0–6.2)	0.045^a^	2.5	(0.7–9.5)	0.17
TLG_total_	1.0	(1.0–1.0)	0.75	1.0	(1.0–1.0)	0.84
Staging (metastatic vs. localized)	2.6	(1.1–6.2)	0.03^a^	1.8	(0.6–6.0)	0.31
Histologic response (poor vs. good)	2.5	(1.0–6.3)	0.047^a^	2.6	(0.7–9.7)	0.17
MTV_total_	1.0	(1.0–1.0)	0.17	1.0	(1.0–1.0)	0.92
Staging (localized vs. metastatic)	2.6	(1.1–6.4)	0.04^a^	1.7	(0.5–5.7)	0.37
Histologic response (good vs. poor)	2.5	(1.0–6.3)	0.045^a^	2.5	(0.7–9.4)	0.19

*CI* confidence interval, *EFS* event-free survival, *HR* hazard ratio, *SUL* standardized uptake value lean-body mass corrected, *OS* overall survival

^a^ Indicates significance

**Fig. 4:**
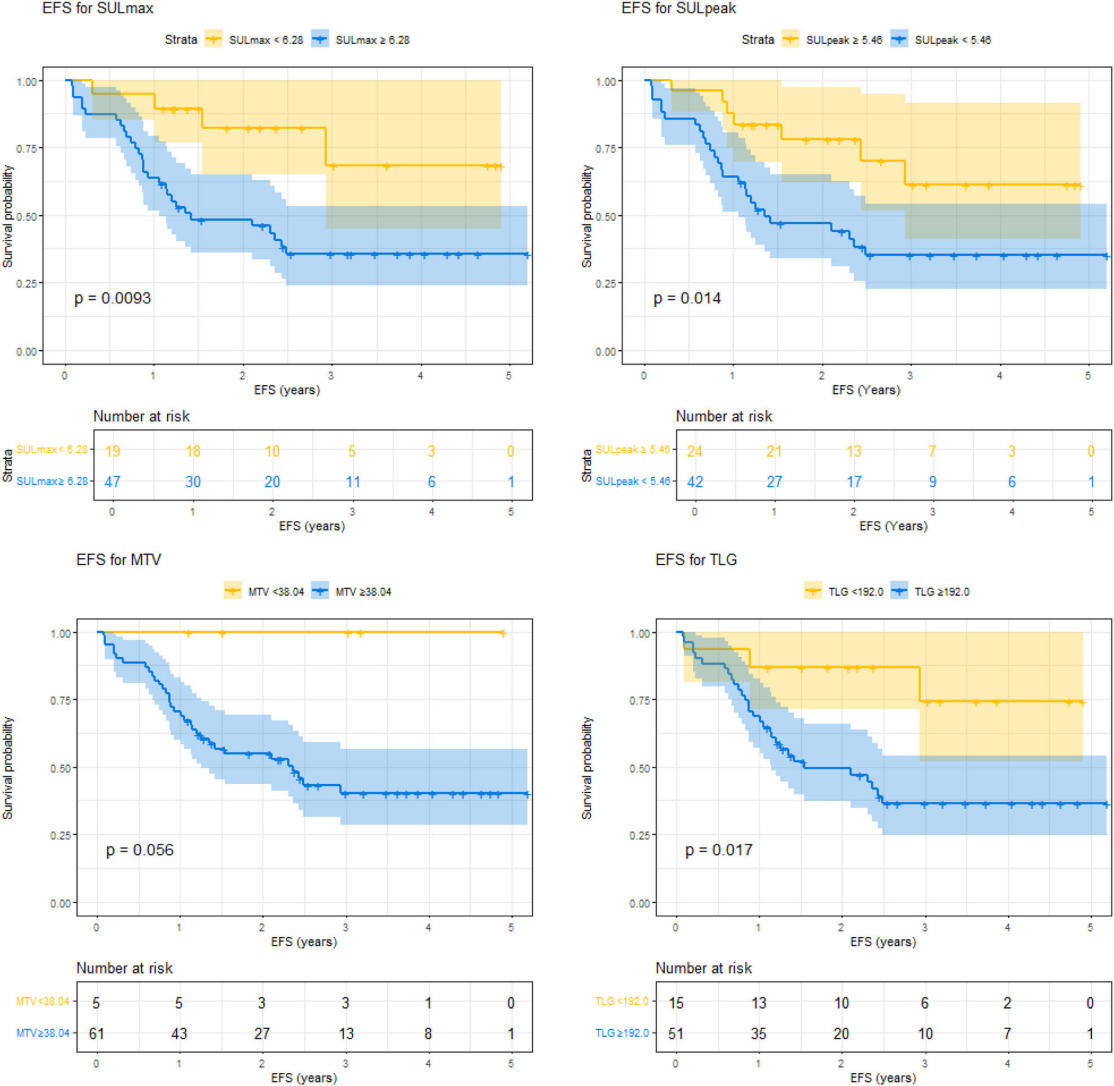
Kaplan–Meier survival plots for event-free survival (EFS) for maximally rank selected values for SUL_max_ (left, above), SUL_peak_ (right, above), MTV_total_ (left, down) and TLG_total_ (right, down)

Cutoffs were calculated for the [^18^F]FDG PET values using the described methods, and log-rank analyses were performed to test the survival probability curves of the defined groups for survival (Table 4 and Fig. 4). ROC curves for event status had an AUC of 0.67, 0.66, 0.64 and 0.64 for SUL_max_, SUL_peak_, MTV_total_ and TLG_total_, respectively (Supplemental Fig. S3). Log-rank analysis showed a significant difference in EFS for all SUL_max_ cutoffs (median-based cutoff: 8.07; max Youden-based cutoff: 8.54; maximally selected rank cutoff: 6.28; published cutoff [18]: 6.0) and SUL_peak_ cutoffs (median-based cutoff: 6.93; max Youden-based cutoff: 6.90; maximally selected rank cutoff: 5.46; published cutoff [16]: 7.98). For MTV_total_, only the maximum Youden cutoff (80.37 cm^3^) resulted in a significant EFS difference. For TLG_total_, the maximum Youden cutoff (193.8) and maximally selected rank cutoff (192.0) resulted in a significant EFS difference. No cutoff for any measure showed a significant difference in OS between the groups. Plots of the distribution of the parameter and the standardized log-rank statistic for maximally selected rank statistics for SUL_max_ and SUL_peak_ showed an equally distinct predictive value for a range of cutoff values. For SUL_max_, any cutoff value between 6 and 10 was equally predictive (Fig. 3a). For SUL_peak_, any cutoff value between 5 and 9 had equal predictive value (Fig. 3b). Cox regression and log-rank subgroup analyses of patients with localized disease did not reveal any other results.

**Table 4:** Log-rank analysis performed for EFS and OS for different cutoffs

	Cutoff method	Cutoff value	EFS log-rank (*p*-value)	OS log-rank (*p*-value)
SUL_max_	Median	8.07	0.01^a^	0.56
	Max Youden	8.54	< 0.01^a^	0.49
	Maximally selected rank	6.28	< 0.01^a^	0.44
	Published cutoff [18]	6	0.04^a^	0.68
SUL_peak_	Median	6.93	0.03^a^	0.90
	Max Youden	6.90	0.03^a^	0.90
	Maximally selected rank	5.46	0.01^a^	0.84
	Published cutoff [16]	7.98	0.02^a^	0.64
MTV_total_	Median (cm^3^)	143.9	0.08	0.64
	Max Youden (cm^3^)	80.37	0.03^a^	0.22
	Maximally selected rank (cm^3^)	38.04	0.06	0.2
	Published cutoff [16] (cm^3^)	238.1	0.94	0.86
TLG_total_	Median	531.1	0.08	0.69
	Max Youden	193.8	< 0.01^a^	0.13
	Maximally selected rank	192.0	0.02^a^	0.17
	Published cutoff [16]	981.97	0.72	0.9

*EFS* event-free survival, *MTV* metabolic tumor volume, *SUL* standardized uptake value lean-body mass corrected, *TLG* total lesion glycolysis, *OS* overall survival

^a^ Indicates significance

## Discussion

This retrospective study analyzed baseline [^18^F]FDG PET-CT PERCIST-like quantitative measurements of EARL-accredited reconstructions in pediatric and adolescent patients diagnosed with high-grade osteosarcoma. SUL_peak_, TLG_total_, and MTV_total_ were not associated with EFS or OS in Cox regression analysis in this population. However, Martingale residual plots showed an excessive risk for EFS for higher SUL_max_ and SUL_peak_ values, with a large 95% CI. The standardized log-rank statistic for the maximally selected rank statistics revealed a wide interval for cutoffs of equal performance in SUL_max_ and SUL_peak_. Dichotomization by applying multiple statistical methods from the literature produced different cutoffs for SUL_max_ and SUL_peak_, and all cutoffs provided significant results in the log-rank analysis for EFS, but none for OS. For MTV_total_ and TLG_total_, both significant and non-significant cutoffs were observed for the EFS. Although dichotomization provided significant results, SUL_peak_, TLG_total_, and MTV_total_ were not prognostic in this study, as no association was observed in the Cox regression analysis, in line with the overlapping CIs observed in the Kaplan–Meier analysis and poor AUCs for event status.

General EFS and OS of the full cohort are in a similar range as previous studies [4, 7, 8]. The absence of prognostic value of SUL_max_ and SUL_peak_ is in line with multiple previous studies [20, 23, 26] (Supplemental Table S4), but in contrast to the results of Im et al [16, 17] and Palmerini et al [18]. SUV_max_ was considered a prognostic factor in the study by Palmerini et al in the full cohort of patients by multivariable analysis. However, log-rank analysis of SUV_max_ in patients with osteosarcoma was not significant [18]. The study of Palmerini et al differed from this study on patient inclusion and analysis, which might explain the different results. The study included both pediatric and adult patients with localized bone sarcomas of the extremities. In multivariable analysis, patients with Ewing sarcoma and osteosarcoma were collectively analyzed. The analysis was based on a dichotomous analysis of SUV_max_ in Cox regression with a cutoff of 6. No analysis of SUV_max_ as a continuous variable for the subgroup of patients with osteosarcoma was presented, which might provide a better comparison to this study. The predictive values of MTV (HR 5.0, 95% CI: 1.5–16.8, *p* = 0.046) and TLG (HR 5.7, 95% CI: 1.3–24.5), as reported by Im et al [16], could not be confirmed in this cohort. Both Im et al [16] and this study included only pediatric and adolescent patients with osteosarcoma. Although in this study cohort, the total number of patients and the number of patients with metastatic disease were slightly higher (*n* = 66 vs. *n* = 34 [16] and 32% vs. 25% [16], respectively), no differences in the selected cohorts can clearly explain the different results.

SUV_peak_ at baseline, specifically in the study by Im et al [16] was concluded to be of predictive value based on Kaplan–Meier analysis with cutoff calculated by the maximum Youden index. The calculated cutoff values in this study, as determined by the maximum Youden index, were in a range similar to previously reported SUV_peak_ and SUV_max_ values [16]. However, for MTV_total_ and TLG_total_ cutoffs as calculated by the maximum Youden index were much lower in this study cohort in comparison with cutoffs reported by Im et al (MTV_total_ 80.37 cm^3^ vs. 238.1 cm^3^, TLG_total_ 193.8 vs. 981.97), which might reflect the distribution and question the value of a specific cutoff. In this study, the primary cutoff was calculated using the maximally selected standardized log-rank statistic, as, in contrast to the maximum Youden index, the time component was addressed in this calculation [37]. In the curves displaying the standardized log-rank statistic, a range of values was observed with near-equal predictive values for SUL_max_ and SUL_peak_, which can also be observed in the CI in the Martingale residual plots. The range of near-equal predictive cutoff values observed in this study aligns with the multiple reported predictive cutoff values. The results of this study highlight the limitations of a specific cutoff for clinical use defined by a limited study population. Hypothetically, by increasing the number of patients, the range might become narrower, or a gray non-discriminative zone of values might be identified with a lower cutoff point vs. a higher cutoff point, which in this study was observed to be significantly different for SUL_max_ values equal to or lower than 6 compared to 10 or higher.

Furthermore, the differences in the results might also be explained by technical factors and not by the selected cohort of patients. In the studies by Im et al [16] and Palmerini et al [18], no standardized reconstructions or reference standards have been reported. Higher administered activities and longer acquisition times were used in Im et al (resp. 5.4MBq/kg and 5 min/bed position) [16] and Palmerini et al (resp. 3.7 MBq/kg and 2 min/bed position) [18], compared to this study (resp. 2.0 MBq/kg and 1–2 min/bed position). These differences are also PET-scanner and physician dependent. Furthermore, Im et al [16] performed MTV analysis with a SUV2.5 cutoff, in contrast to this study that used the PERCIST-based liver reference methodology. As identified in the literature, technical, biological, and physical factors, including image reconstruction and the normalization factor of SUV, can significantly affect [^18^F]FDG quantification within a study and hamper harmonization between studies [41]. The specific impact of this variability on the results of studies investigating osteosarcoma is unclear.

To anticipate future harmonization, adherence to EARL/EANM guidelines [28, 34] is essential in [^18^F]FDG PET-CT whole-body staging of high-grade osteosarcoma. Although guidelines have been published for acquisition and analysis [28, 30, 32, 34], only one study reported adherence to quality standards [25] (Supplemental Table S4), which limits the comparison of data between studies. The data presented in this study provide an opportunity for future data harmonization, as data acquisition and analysis were performed according to the EANM guidelines, version 1.0 [34]. Second, only high-risk osteosarcoma patients were included, whereas other bone sarcoma patients were deliberately excluded for a more homogenous study population. To the best of our knowledge, this is the first study to report standardized data in this population according to these guidelines. Despite these strengths, several limitations and points of discussion must be addressed. In addition to being a retrospective cohort, the cohort size was small (as all available published cohorts on osteosarcoma) and insufficient to perform a multivariable analysis that included all currently known prognostic clinical factors. Second, the follow-up time was relatively short, which limited the OS analysis. Furthermore, in this study, only [^18^F]FDG PET-CT at diagnosis was available because response assessment with [^18^F]FDG PET-CT is currently not the standard of care.

## Conclusion

Baseline EARL-standardized [^18^F]FDG PET SUL_peak_, MTV, and TLG were not associated with EFS or OS in a cohort of pediatric and adolescent patients with high-grade osteosarcoma. The proposed prognostic cutoffs of [^18^F]FDG PET-CT from earlier studies could not be independently validated. Future studies should preferably be performed prospectively and internationally for a larger population and, at a minimum, adhere to accredited reconstructions (e.g., EARL or QIBA) to facilitate data comparison and harmonization.

## Supplementary information

ELECTRONIC SUPPLEMENTARY MATERIAL

## Data Availability

Data generated or analyzed during the study are available from the corresponding author upon request.

## Abbreviations

CI: Confidence interval
EARL: European Association of Nuclear Medicine Research Ltd.
EFS: Event-free survival
[^18^F]FDG PET-CT: [^18^F]Fluorodeoxyglucose positron emission tomography-computed tomography
HR: Hazard ratio
MTV: Metabolic tumor volume
OS: Overall survival
SUL: Standardized uptake value lean body mass corrected
TLG: Total lesion glycolysis
