# Supplementary material for: The prognostic value of baseline EARL standardized FDG PET indices in pediatric and adolescent high-grade osteosarcoma
Source: Eur Radiol. 2025 Jan 24;35(7):4233–42. doi: 10.1007/s00330-025-11372-z (PMC12166021; doi:10.1007/s00330-025-11372-z)
Supplement: Supplementary file 1 — ELECTRONIC SUPPLEMENTARY MATERIAL [file 330_2025_11372_MOESM1_ESM.pdf]

The prognostic value of baseline EARL standardized FDG PET indices in pediatric and adolescent high-grade osteosarcoma

ELECTRONIC SUPPLEMENTARY MATERIAL

Table of Contents:

Supplemental Table S1. Baseline characteristics for histologic response
Supplemental Table S2. [^{18}F]FDG PET measurements for histologic response
Supplemental Table S3. [^{18}F]FDG PET measurements for event status
Supplemental Table S4. Available studies investigating the value of FDG PET indices at diagnosis

Supplemental Figure S1. Martingale residual plot with lowess smooth, which displays excessive risk for event (y-axis) in relation to the log10-transformed continuous covariate (x-axis) data (MTV – upper figure, TLG – lower figure). Both parameters show a nonlinear relation a with event risk

Supplemental Figure S2. Distribution by class and standardized log-rank statistic for maximally selected rank statistics (MTV – upper figure, TLG – lower figure)

Supplemental Figure S3. ROC curves for SUL_{max}, SUL_{peak}, MTV_{total} and TLG_{total} for event status

Eur Radiol (2025) van Ewijk R, Dandis R, Rodewijk J, et al.

Supplemental Table S1. Baseline characteristics for histologic response

	Good (N=20)	Poor (N=42)	*p* value
Age (years)			
Mean (SD)	12.4 (3.70)	13.4 (2.98)	0.286
Median [Min, Max]	13.0 [4.00, 17.0]	14.0 [5.00, 18.0]	
Sex			
Male	12 (60.0%)	28 (66.7%)	0.819
Female	8 (40.0%)	14 (33.3%)	
Tumor primary site			
Lower extremity	19 (95.0%)	37 (88.1%)	NA
Upper extremity	1 (5.0%)	4 (9.5%)	
Axial	0 (0%)	1 (2.4%)	
Other	0 (0%)	0 (0%)	
Primary tumor size (cm)			
<8	4 (20.0%)	8 (19.0%)	1
>8	16 (80.0%)	34 (81.0%)	
Staging			
Localized	11 (55.0%)	32 (76.2%)	0.0177
Metastatic - Pulmonary only	9 (45.0%)	6 (14.3%)	
Metastatic	0 (0%)	4 (9.5%)	
Margins			
Negative	20 (100%)	41 (97.6%)	1
Positive	0 (0%)	1 (2.4%)	
Event status			
No	13 (65.0%)	19 (45.2%)	0.237
Yes	7 (35.0%)	23 (54.8%)	
Patient status			
Alive	17 (85.0%)	28 (66.7%)	0.899
Death	3 (15.0%)	14 (33.3%)	

Supplemental Table S2. [^{18}F]FDG PET measurements for histologic response

	Good (N=20)	Poor (N=42)	*p* value
SUL_{peak}			
Mean (SD)	7.73 (4.17)	7.77 (3.38)	0.966
Median [Min, Max]	6.62 [3.38, 20.3]	7.77 [2.91, 19.7]	
SUL_{max}			
Mean (SD)	9.09 (5.01)	9.38 (4.02)	0.821
Median [Min, Max]	7.78 [4.72, 25.4]	9.08 [3.88, 22.5]	
Total.body.MTV.liver			
Mean (SD)	148 (99.8)	152 (98.7)	0.901
Median [Min, Max]	145 [14.7, 346]	142 [1.67, 409]	
Total.body.TLG.Liver			
Mean (SD)	607 (457)	611 (433)	0.97
Median [Min, Max]	609 [45.1, 1630]	540 [4.68, 1770]	

Supplemental Table S3. [^{18}F]FDG PET measurements for event status

	0<br>(N=33)	1<br>(N=33)	*p* value
SUL_{peak}			
Mean (SD)	7.14 (4.32)	8.10 (2.61)	0.279
Median [Min, Max]	5.36 [2.91, 20.3]	8.72 [3.62, 12.3]	
SUL_{max}			
Mean (SD)	8.54 (5.12)	9.75 (3.14)	0.252
Median [Min, Max]	6.51 [3.88, 25.4]	10.2 [4.12, 15.3]	
MTV_{total}			
Mean (SD)	132 (106)	170 (86.6)	0.11
Median [Min, Max]	109 [1.67, 409]	167 [38.5, 346]	
TLG_{total}			
Mean (SD)	521 (472)	680 (381)	0.138
Median [Min, Max]	386 [4.68, 1770]	752 [119, 1630]	

Supplemental Table S4. Available studies investigating the value of FDG PET indices at diagnosis

	n	Age (years)	Histology	FDG PET values	Study Endpoint	Prognostic	Adjustment (BW or LBM)	Parameters in multivariable analysis	SUVmax	Prognostic cut-off	Design	Quality standard
Im 2018(18) / Davis 2018 (19)	34	Median 12<br>Range 7 - 19	Osteosarcoma	SUVmax,<br>SUVpeak<br>MTV<br>TLG	EFS, OS	No; No<br>No; No<br>Yes; Yes<br>Yes; Yes	Not reported	SUVmax, SUVpeak, MTV (SUV2.5), TLG (SUV2.5), TLG(SUV2.5), MTV (liver +2SD), TLG (liver + 2SD), histologic response, metastasis	Mean 11.7<br>Range 3.7 – 30.3	SUVpeak 7.97<br>MTV2.5 236.06<br>TLG2.5 981.97	Prospective, single center	Not reported
Palmerini 2017 (20)	77	Mean 17<br>Range 3 – 39	Bone sarcoma	SUVmax	EFS	Yes	Not reported	Histology (osteo/ewing), SUV1 (low/high), LDH (low/high), ALP (low/high)	Median 7.85<br>Range 0 - 24	SUVmax 6	Retrospective, single center	Not reported
Byun 2015 (21)	31	Median 15<br>Range 14 – 21	Osteosarcoma	SUVmax<br>RImax<br>RImean	HR	No<br>No<br>Yes	BW	Not performed	Mean 9.40<br>Range 5.8 – 10.9	-	Prospective, single center	Not reported
Byun 2013 (22)	83	Median 16<br>Range 8 – 37	Osteosarcoma	SUVmax<br>MTV<br>TLG	MFS	No<br>Yes<br>Yes	BW	SUVmax, MTV, TLG, age, sex, AJCC stage, tumor location, histologic subtype, and histologic response	Mean 9.5<br>SD 5.7	MTV 105	Retrospective, single center	Not reported
Kong 2013 (23)	26	Mean 21<br>Range 9 – 55	Osteosarcoma	SUVmax	HR	No	BW	Not performed	Mean 9.2<br>Range 2.9 – 31.2	-	Prospective, single center	Not reported
Bajpai 2011 (24)	31	Median 17	Osteosarcoma	SUVmax	HR	No	Not reported	Not performed	Mean 6.3<br>Range 2.3 – 15.7	-	Prospective, single center	Not reported
Hawkins 2009 (25)	40	Median 15<br>Range 7 – 31	Osteosarcoma	SUVmax	PFS	No	Not reported	Not performed	Median 6.8<br>Range 3.0 – 24.1	SUVmax 6	Retrospective, multicenter	Not reported
Cheon 2009 (26)	70	Median 14<br>Range 5 – 59	Osteosarcoma	SUVmax	HR	No	BW	Not performed	Median 8.0<br>Range 2.4 – 47.5	-	Prospective, single center	Not reported
Costelloe 2009 (27)	34	Mean 27<br>Range 9 – 65	Osteosarcoma	SUVmax<br>TLG	PFS; OS	Yes; No<br>No; Yes	LBM	Not performed	Mean 10.6<br>Range 3.2 – 36.1	SUVmax 15	Retrospective, single center	NCI standard (31)
Eary 2008 (28)	234	Median 45.3<br>Range 18 – 86	Bone sarcoma / Soft tissue sarcoma	SUVmax<br>Heterogeneity	DFS, OS	No; No<br>Yes; Yes	Not reported	Stage, age, sex, grade, subtype, size, heterogeneity	Mean 8.2<br>Range 1.5 – 40.3	-	Retrospective, single center	Not reported
Franzius 2002 (29)	29	Median 14<br>Range 5 – 41	Osteosarcoma	T/NT $ratio_{max}$<br>T/NT $ratio_{max}$	EFS<br>OS	Yes<br>Yes	Not reported	Not performed	NA	-	Retrospective, single center	Not reported

BW: Body weight, EFS: Event-free survival, HR: Histologic response, LBM: Lean body mass, MTV: Metabolic tumor volume, MFS: Metastasis-free survival, NA: not available, PFS: progression-free survival, T/NT: Tumor/non-tumor, TLG: Total lesion glycolysis, OS: Overall survival, SUV: Standard uptake value

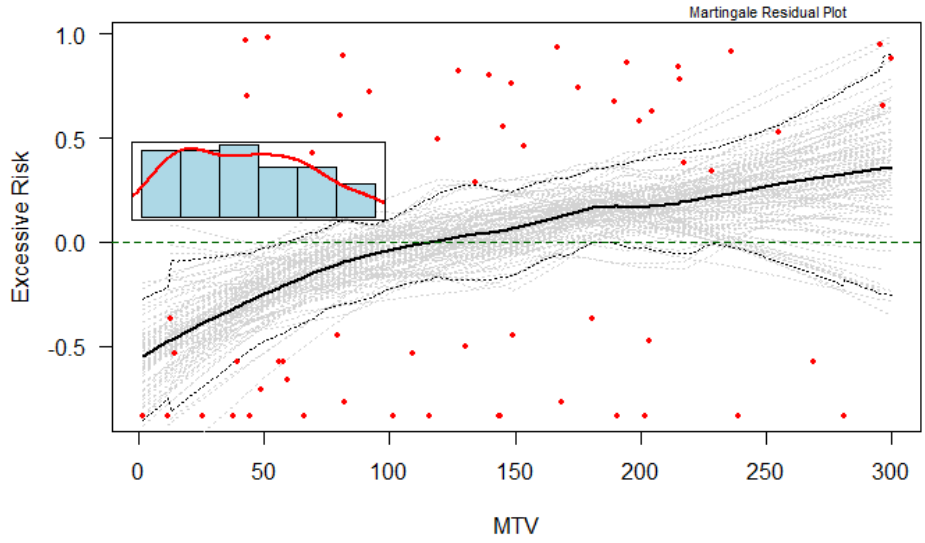

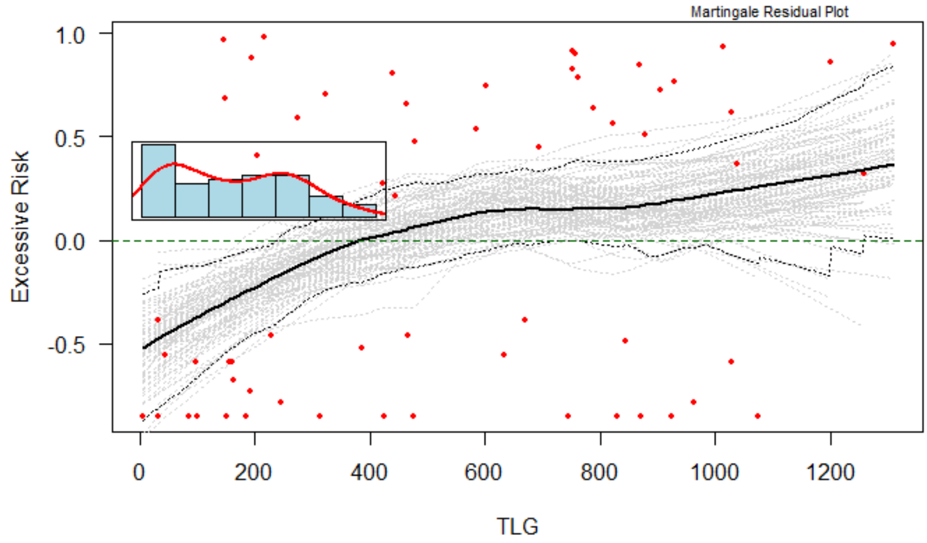

Supplemental Figure S1. Martingale residual plot with lowess smooth, which displays excessive risk for event (y-axis) in relation to the continuous covariate (x-axis) data (MTV – upper figure, TLG – lower figure). Both parameters show a nonlinear relation a with event risk (EFS).

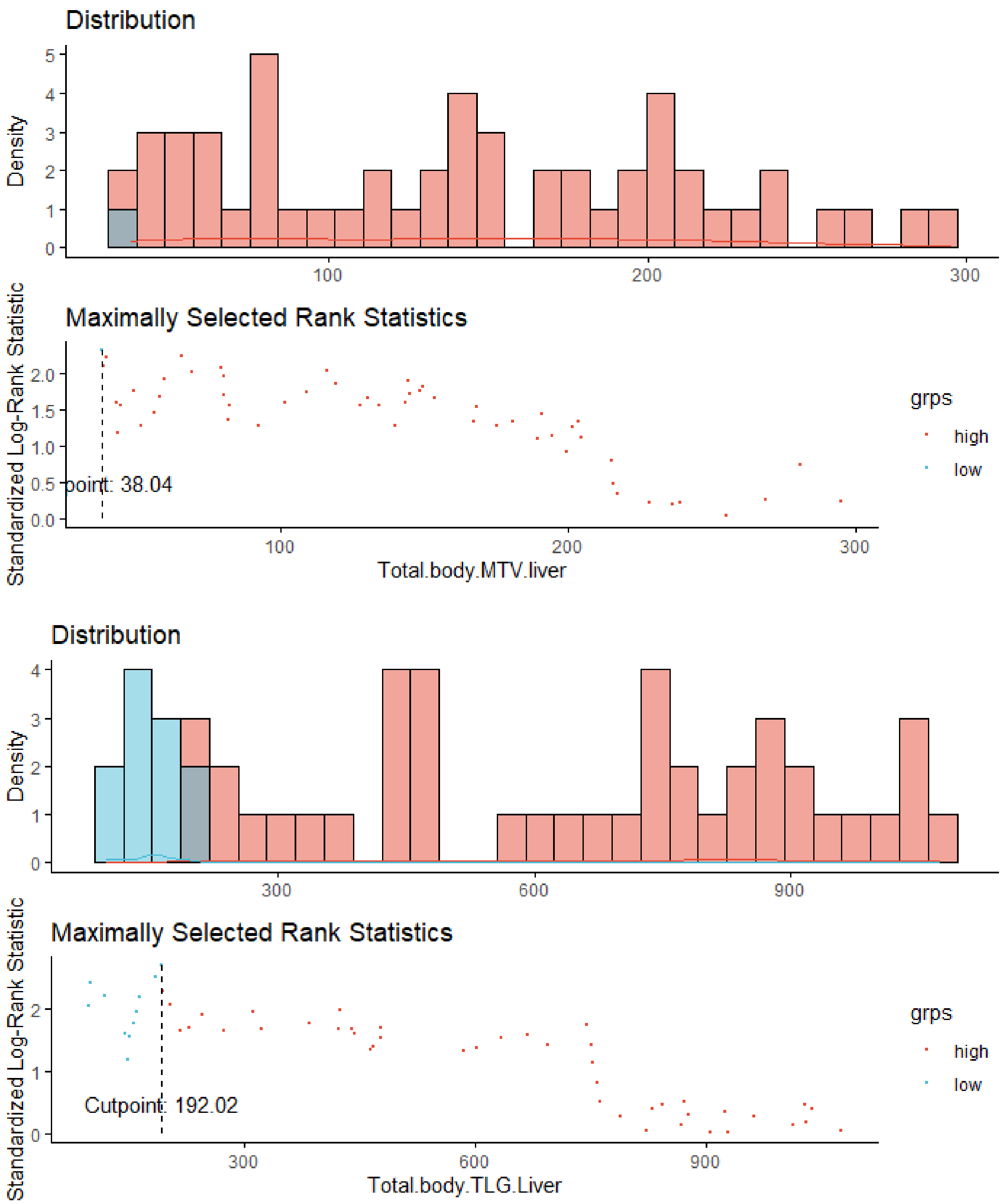

Supplemental Figure S2. Distribution by class and standardized log-rank statistic for maximally selected rank statistics (MTV – upper figure, TLG – lower figure).

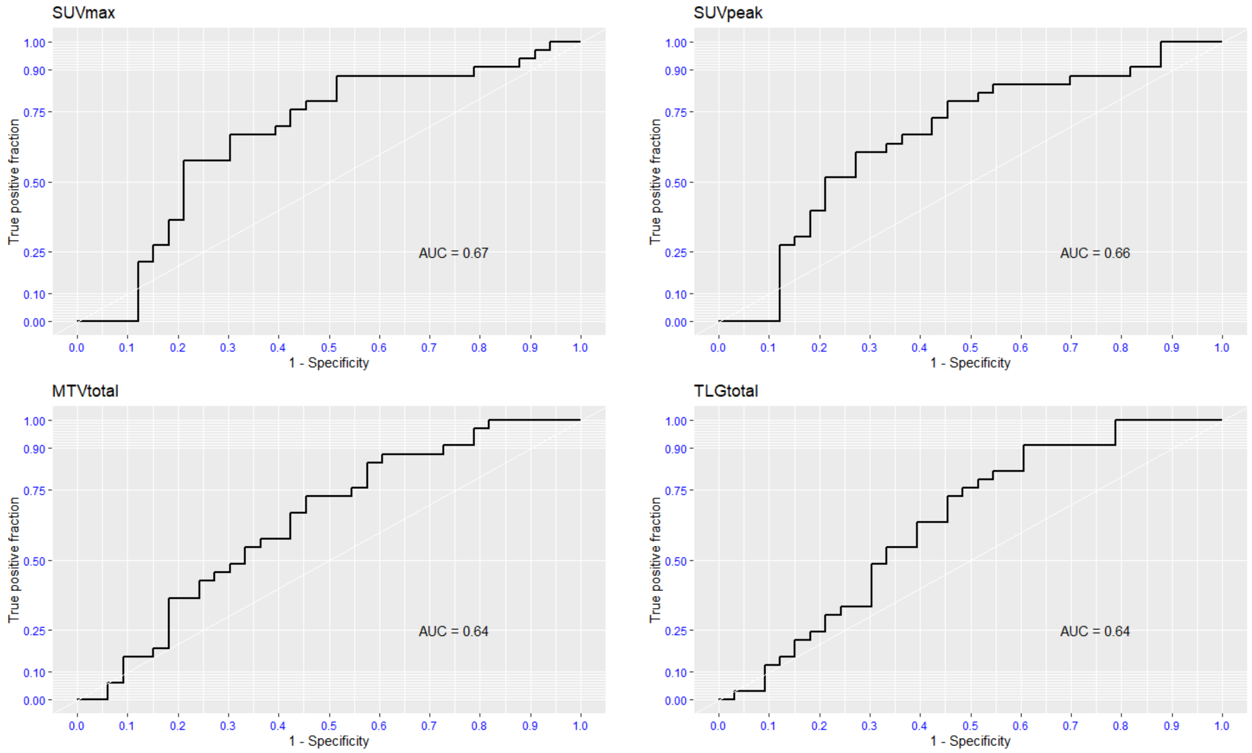

Supplemental Figure S3. ROC curves for SUL_{max}, SUL_{peak}, MTV_{total} and TLG_{total} for event status
